# Dietary acrylamide and cancer of the large bowel, kidney, and bladder: Absence of an association in a population-based study in Sweden

**DOI:** 10.1038/sj.bjc.6600726

**Published:** 2003-01-28

**Authors:** L A Mucci, P W Dickman, G Steineck, H-O Adami, K Augustsson

**Affiliations:** 1 Department of Medical Epidemiology, Karolinska Institutet, Box 281, SE 171 77 Stockholm, Sweden; 2 Department of Epidemiology, Harvard School of Public Health, 677 Huntington Avenue, 9th floor, Boston, MA 02115, USA; 3 Department of Oncology and Pathology, Clinical Cancer Epidemiology, Karolinska Institutet, SE 171 76 Stockholm, Sweden

**Keywords:** acrylamide, kidney cancer, large bowel cancer, bladder cancer, diet

## Abstract

Recently, disturbingly high levels of acrylamide were unexpectedly detected in widely consumed food items, notably French fries, potato crisps, and bread. Much international public concern arose since acrylamide has been classified as a probable carcinogen, although based chiefly on laboratory evidence; informative human data are largely lacking. We reanalysed a population-based Swedish case–control study encompassing cases with cancer of the large bowel (*N=*591), bladder (*N=*263) and kidney (*N=*133), and 538 healthy controls, assessing dietary acrylamide by linking extensive food frequency data with acrylamide levels in certain food items recorded by the Swedish National Food Administration. Unconditional logistic regression was used to estimate odds ratios, adjusting for potential confounders. We found consistently a lack of an excess risk, or any convincing trend, of cancer of the bowel, bladder, or kidney in high consumers of 14 different food items with a high (range 300–1200 *μ*g kg^−1^) or moderate (range 30–299 *μ*g kg^−1^) acrylamide content. Likewise, when we analysed quartiles of known dietary acrylamide intake, no association was found with cancer of the bladder or kidney. Unexpectedly, an inverse trend was found for large bowel cancer (*P* for trend 0.01) with a 40% reduced risk in the highest compared to lowest quartile. We found reassuring evidence that dietary exposure to acrylamide in amounts typically ingested by Swedish adults in certain foods has no measurable impact on risk of three major types of cancer. It should be noted, however, that relation of risk to the acrylamide content of all foods could not be studied.

Acrylamide is classified by the International Agency for Research on Cancer (IARC) as a probable human carcinogen (IARC, 1994). In April 2002, a Swedish food survey created international public health alarm by its report of substantially elevated levels of acrylamide in frequently consumed food products (Swedish National Food Administration (NFA), 2002) and in particular, in potato crisps, French fries, and crisp bread. Acrylamide formation probably occurs as a result of a reaction between amino acids and reducing sugars during the heating of starch-rich foods to high temperatures (Mottram *et al*, 2002; Stadler *et al*, 2002).

Although animal models support a dose–response relation between acrylamide and cancer at multiple sites (Bull *et al*, 1984; Johnson *et al*, 1986), no epidemiological study to date has examined whether higher intake of foods containing high acrylamide level increases the risk of any cancer. The Swedish NFA findings therefore led to much public concern in Europe and the United States. In response, we have analysed data from a population-based case–control study in Sweden to investigate whether higher intake of certain food items with higher acrylamide content increases the risk of cancers of the large bowel, bladder, or kidney.

## MATERIALS AND METHODS

### Study population

This investigation was undertaken in an existing population-based case–control study of a possible relation between heterocyclic amines in fried foods, and cancers of the large bowel and urinary tract (Augustsson *et al*, 1999). The study base comprised individuals born in Sweden between 1918 and 1942, and residing in Stockholm for at least 1 month between November 1992 and December 1994. Incident cases were identified in the Stockholm area from the population-based cancer registry and included the following sites: large bowel (ICD9 153 and 154), bladder (ICD9 188), and kidney (ICD9 189, excluding renal pelvis 189.1 and 189.3). Controls were randomly selected from the Register of Total Population during the study period, and frequency matched by age and gender.

Survey questionnaires were mailed to 692 controls, and 875, 391, 186 cases of cancer of the large bowel, bladder, and kidney, respectively. As previously described (Augustsson *et al*, 1999), participation rates were 80% for controls, and between 68 and 74% for cases. Reasons for nonparticipation included death, illness, refusal, or loss of questionnaire. An additional 40 respondents (15 controls and 25 cases) had missing dietary data on >10% of items and were dropped from the analysis. The sample size for the current study was 538 controls, and 591, 263, 133 cases with cancer of the large bowel, bladder and kidney, respectively. This study was approved by the Ethical Committee of Karolinska Institutet.

### Dietary data

Dietary habits in the 5 years prior to the study were assessed by semiquantitative food frequency questionnaire of 188 food items. Respondents could choose from 10 intake frequency categories ranging from two to three times per day to never.

In addition, more than 100 food samples were analysed to determine acrylamide levels at the NFA. The majority of the foods found to contain high levels of acrylamide were covered by the food frequency questionnaire including French fries, potato crisps, fried potatoes, crisp bread, breakfast cereals, and biscuits. A summary of median acrylamide levels is given in Table 1

**Table 1:** Acrylamide concentration for several food groups: Swedish National Food Administration, 2002

	**Acrylamide concentration (***μ***g kg^−1^)**		
**Food group**	**Median**	**Min–max**	**Number of samples**
Potato crisps	1200	330–2300	14
French fries	450	300–1100	9
Pan-fried potatoes	300		1
Biscuits and crackers	410	<30–650	14
Crisp breads	140	<30–1900	21
Breakfast cereals	160	<30–1400	15
Corn chips	150	120–180	3
Soft breads	50	<30–160	20
Various fried foods (pizza, pancakes, waffles, fish fingers, meatballs, chickenbits, deep fried fish, vegetarian schnitzel, and cauliflower gratin)	40	<30–60	9

. The survey found that foodstuffs that were not fried, deep fried, or oven-baked during production or preparation did not contain any appreciable levels of acrylamide.

A summary measure of acrylamide dose was determined by ranking the following food items on acrylamide dose: potato crisps, French fries, fried potatoes, fried pancakes, pizza, meatballs, breaded fish, cereals, crisp and soft bread, and biscuits. The foodstuffs were ranked according to the median acrylamide content determined by the Swedish NFA as follows: food stuffs without appreciable acrylamide were assigned a rank of 0; food stuffs with a median acrylamide content less than 100 *μ*g kg^−1^ were assigned a rank of 1; those with a median acrylamide dose 100–200 *μ*g kg^−1^ were assigned a rank of 2; those with a median acrylamide dose 200–600 *μ*g kg^−1^ were assigned a rank of 4; and those with a median acrylamide dose greater than 600 *μ*g kg^−1^ were assigned a rank of 8. We converted an individual's intake of each food item to grams per day, multiplied this by the acrylamide level ranking for the specific food item, and summed up the ranks across all of the above foods items for each individual.

### Statistical analysis

We assessed the risk of each cancer separately for 14 food items found to contain high acrylamide levels (median >40 *μ*g kg^−1^) and for the total summary measure of dietary acrylamide. Unconditional logistic regression was employed to calculate odds ratios (OR), an estimate of the rate ratio, and 95% confidence intervals (95% CI).

Quartiles of the summary acrylamide measure were created based on the distribution of the control group, and modelled as categorical variables with the lowest quartile as referent group. Breads were modelled as servings per day. Tests for trend were calculated using likelihood ratio tests, where the categorical medians of each quartile were modelled as ordinal covariates. In multivariate models, we considered as potential confounders smoking (current, former, never), body mass index (categorical), alcohol intake (categorical-colorectal cancer only), fruit and vegetable intake (continuous), saturated fat density (quintiles, categorical), red meat density (quintiles, categorical), and total energy (log transformed). Variables that were statistically significant at *α*=0.20, or which confounded the relation between acrylamide and cancer, were included in the final model (Rothman and Greenland, 1998). Since smoking contributes appreciably to acrylamide exposure, we examined possible differences in cancer risk among smokers and nonsmokers through stratification and through adding interaction term(s) in the models.

Finally, a sensitivity analysis was performed to examine the extent of misclassification by using the median acrylamide concentrations to rank food items and calculate total dietary acrylamide dose. In this approach, we calculated summary measures by reranking individual food items based alternatively on the minimum and maximum acrylamide content measured in the Swedish NFA samples. All statistical analyses were performed using SAS version 8.2.

## RESULTS

Lifestyle and dietary characteristics of cases and controls are shown in Table 2

**Table 2:** Distribution of demographic, lifestyle and dietary factors among controls and cases of large bowel, bladder and kidney cancer

	**Controls**	**Cancer cases of large bowel**	**Cancer cases of bladder**	**Cancer cases of kidney**
	***N (%)***	***N (%)***	***N (%)***	***N (%)***
**Characteristics of participants**				
*Gender*				
Males	272 (50.6%)	346 (58.5%)	199 (75.7%)	71 (53.4%)
Females	266 (49.4%)	245 (41.5%)	64 (24.3%)	62 (46.6%)
*Age group (years)*				
51–62	141 (26.2%)	167 (28.3%)	54 (20.5%)	45 (33.8%)
63–68	130 (24.2%)	137 (23.2%)	77 (29.3%)	30 (22.6%)
69–72	137 (25.5%)	137 (23.2%)	64 (24.3%)	35 (26.3%)
73–77	130 (24.2%)	150 (25.4%)	68 (25.9%)	23 (17.3%)
Body mass index (kg m^−2^) >25.0	205 (38.5%)	316 (53.8%)	140 (53.5%)	70 (53.4%)
Current smokers	130 (24.2%)	134 (22.7%)	128 (48.7%)	34 (25.6%)
				
**Food intake 5 years prior**				
*Weekly consumption of*				
* *French fries	33 (6.1%)	58 (9.8%)	23 (8.8%)	36 (34.6%)
* *Potato crisps	54 (10.0%)	48 (8.1%)	14 (5.3%)	16 (11.3%)
Pan-fried potatoes	202 (37.6%)	226 (38.2%)	124 (47.2%)	49 (36.8%)
Potatoes au gratin	42 (7.8%)	59 (10.0%)	17 (6.5%)	15 (11.3%)
Cornflakes	186 (34.6%)	161 (27.2%)	66 (25.1%)	31 (23.3%)
Muesli	172 (32.4%)	168 (28.8%)	70 (27.0%)	42 (31.8%)
Oatmeal	244 (45.3%)	211 (35.8%)	88 (33.5%)	46 (34.6%)
Biscuits	257 (47.8%)	302 (51.2%)	123 (47.0%)	66 (49.6%)
Fried meatballs	54 (10.0%)	50 (8.5%)	27 (10.3%)	19 (14.3%)
Breaded fish	100 (19.5%)	132 (23.7%)	55 (22.1%)	30 (24.0%)
Pancakes/waffles	181 (33.6%)	193 (32.7%)	80 (30.4%)	43 (32.3%)
*3+ slices per day of*				
Crisp bread	104 (21.2%)	117 (19.8%)	57 (12.2%)	28 (31.0%)
Rye bread	45 (8.3%)	63 (10.6%)	35 (13.3%)	15 (11.3%)
White bread	54 (10.0%)	61 (10.3%)	26 (9.9%)	20 (15.0%)
				
**Other dietary factors**	**Mean (SE)**	**Mean (SE)**	**Mean (SE)**	**Mean (SE)**
Fruit and vegetables (g day^−1^)	275.3 (8.2)	250.1 (6.8)	234.9 (11.1)	260.6 (15.2)
Saturated fat (g day^−1^)	35.5 (0.7)	38.1 (0.7)	40.4 (1.3)	36.8 (1.5)
Total calories (kcal day^−1^)	2065.9 (27.5)	2186.4 (29.3)	2243.0 (49.4)	2150.3 (62.9)
Alcohol (g day^−1^)	10.8 (0.6)	11.8 (0.6)	15.2 (1.1)	10.8 (1.4)
				
**Acrylamide intake through diet (***μ***g day^−1^)**	27.5 (0.6)	28.6 (0.6)	29.4 (0.9)	28.4 (1.2)

. Weekly intake of French fries and potato crisps by both cases and controls was low, while intake of pan-fried potatoes was more common. About one-fifth of controls consumed three or more slices of crisp bread per day. These data suggest that bread products and pan-fried potatoes account for a major source of dietary acrylamide. Estimated mean daily intake of acrylamide through diet was 27.5 *μ*g among controls, based on foodstuffs investigated by the Swedish NFA as of April 2002.

The association between foodstuffs containing high acrylamide levels and risk of cancers of the large bowel and urinary tract is presented in Table 3a

**Table 3:** Consumption of foods with elevated levels of acrylamide, and risk of cancer of the large bowel, bladder and kidney

	**Cancer of large bowel**		**Cancer of bladder**		**Cancer of kidney**	
	**Adjusted^a^**	**Adjusted^b^**	**Adjusted^a^**	**Adjusted^b^**	**Adjusted^a^**	**Adjusted^b^**
	**OR (95% CI)**	**OR (95% CI)**	**OR (95% CI)**	**OR (95% CI)**	**OR (95% CI)**	**OR (95% CI)**
Potatoes						
*French Fries (high levels)*						
Never	1 (Ref)	1 (Ref)	1 (Ref)	1 (Ref)	1 (Ref)	1 (Ref)
Less often	1.3 (1.0–1.7)	1.3 (0.9–1.7)	1.1 (0.7–1.5)	0.8 (0.5–1.3)	0.9 (0.6–1.5)	0.9 (0.5–1.4)
2–3 times per month	0.9 (0.6–1.3)	0.8 (0.6–1.2)	0.8 (0.5–1.4)	1.0 (0.6–1.6)	0.9 (0.5–1.6)	0.7 (0.4–1.4)
Weekly	0.9 (0.6–1.5)	0.8 (0.5–1.4)	0.7 (0.4–1.3)	0.7 (0.4–1.1)	0.9 (0.4–1.8)	0.7 (0.3–1.6)
*Potato crisps (high levels)*						
Never	1 (Ref)	1 (Ref)	1 (Ref)	1 (Ref)	1 (Ref)	1 (Ref)
Less often	1.3 (1.0–1.7)	1.3 (1.0–1.7)	1.2 (0.9–1.7)	1.2 (0.8–1.7)	1.6 (1.1–2.4)	1.7 (1.1–2.6)
2–3 times per month	0.9 (0.6–1.4)	0.8 (0.5–1.3)	1.2 (0.7–2.1)	1.3 (0.7–2.3)	0.9 (0.4–1.9)	0.9 (0.4–1.9)
Weekly	1.4 (0.8–2.2)	1.3 (0.8–2.1)	0.9 (0.5–1.8)	0.9 (0.4–1.8)	0.8 (0.3–2.0)	0.7 (0.3–1.9)
*Pan*-*Fried Potatoes (high levels)*						
Never	1 (Ref)	1 (Ref)	1 (Ref)	1 (Ref)	1 (Ref)	1 (Ref)
Less often	1.0 (0.7–1.6)	1.0 (0.6–1.5)	1.1 (0.6–2.0)	1.1 (0.5–2.1)	0.8 (0.4–1.6)	0.7 (0.4–1.5)
2–3 times per month	1.0 (0.6–1.6)	0.9 (0.5–1.4)	1.2 (0.6–2.3)	1.2 (0.6–2.3)	0.9 (0.5–1.8)	0.8 (0.4–1.7)
Weekly	1.0 (0.6–1.5)	0.8 (0.5–1.2)	1.4 (0.8–2.5)	1.3 (0.7–2.4)	0.8 (0.4–1.6)	0.7 (0.4–1.4)
*Potatoes au Gratin (unknown levels)*						
Never	1 (Ref)	1 (Ref)	1 (Ref)	1 (Ref)	1 (Ref)	1 (Ref)
Less often	1.1 (0.7–1.7)	1.2 (0.8–1.8)	0.7 (0.4–1.1)	0.7 (0.4–1.1)	0.8 (0.4–1.5)	0.8 (0.4–1.5)
2–3 times per month	1.1 (0.7–1.7)	1.2 (0.7–1.9)	0.7 (0.4–1.2)	0.7 (0.4–1.3)	0.8 (0.4–1.5)	0.8 (0.4–1.7)
Weekly	1.3 (0.8–2.3)	1.5 (0.8–2.7)	0.5 (0.2–1.1)	0.5 (0.2–1.0)	1.0 (0.4–2.4)	1.1 (0.4–2.6)
*Total potatoes fried or baked*						
Less often/Never	1 (Ref)	1 (Ref)	1 (Ref)	1 (Ref)	1 (Ref)	1 (Ref)
2–3 per month	1.0 (0.7–1.4)	0.9 (0.6–1.2)	1.2 (0.7–1.9)	1.2 (0.7–2.0)	1.1 (0.6–1.8)	1.0 (0.6–1.8)
Weekly	0.8 (0.6–1.1)	0.7 (0.5–1.0)	1.2 (0.8–1.9)	1.1 (0.7–1.8)	0.9 (0.5–1.4)	0.7 (0.4–1.3)
Daily	1.6 (0.9–2.7)	1.2 (0.6–2.2)	1.7 (0.8–3.4)	1.6 (0.7–3.5)	1.1 (0.4–2.7)	0.8 (0.3–2.2)
						
Bread						
*Crisp bread (moderate levels)*						
None	1 (Ref)	1 (Ref)	1 (Ref)	1 (Ref)	1 (Ref)	1 (Ref)
1–2 slices per day	1.0 (0.7–1.4)	1.0 (0.7–1.4)	0.9 (0.6–1.3)	0.8 (0.5–1.2)	1.0 (0.6–1.7)	0.9 (0.6–1.6)
3 slices per day	0.9 (0.6–1.4)	0.8 (0.5–1.3)	0.7 (0.4–1.2)	0.6 (0.3–1.2)	0.7 (0.7–1.6)	0.6 (0.3–1.5)
4+ slices per day	0.8 (0.5–1.3)	0.7 (0.4–1.1)	0.9 (0.5–1.6)	0.7 (0.4–1.4)	1.3 (0.6–2.6)	1.2 (0.6–2.5)
*Rye bread (moderate levels)*						
None	1 (Ref)	1 (Ref)	1 (Ref)	1 (Ref)	1 (Ref)	1 (Ref)
1–2 slices per day	1.0 (0.8–1.3)	0.9 (0.7–1.2)	0.8 (0.5–1.0)	0.7 (0.5–1.0)	1.0 (0.7–1.5)	0.9 (0.6–1.4)
3 slices per day	1.6 (0.9–3.0)	1.5 (0.8–2.8)	1.3 (0.6–2.7)	1.4 (0.6–3.0)	1.7 (0.7–4.3)	1.7 (0.7–4.5)
4+ slices per day	0.9 (0.5–1.6)	0.7 (0.4–1.3)	1.0 (0.6–2.0)	0.9 (0.5–1.8)	1.3 (0.6–3.0)	1.2 (0.5–2.9)
*White bread (moderate levels)*						
None	1 (Ref)	1 (Ref)	1 (Ref)	1 (Ref)	1 (Ref)	1 (Ref)
1–2 slices per day	1.3 (1.0–1.7)	1.2 (0.9–1.6)	1.2 (0.9–1.7)	1.0 (0.7–1.5)	0.9 (0.6–1.3)	0.8 (0.5–1.3)
3 slices per day	1.5 (0.8–2.7)	1.3 (0.7–2.3)	0.9 (0.4–2.0)	0.7 (0.3–1.8)	1.8 (0.8–4.3)	1.7 (0.7–4.1)
4+ slices per day	0.9 (0.6–1.6)	0.7 (0.4–1.3)	0.9 (0.5–1.8)	0.6 (0.3–1.3)	1.3 (0.6–2.7)	1.0 (0.5–2.3)
Cereal						
*Cornflakes (moderate levels)*						
Never	1 (Ref)	1 (Ref)	1 (Ref)	1 (Ref)	1 (Ref)	1 (Ref)
Less often	1.5 (1.1–2.0)	1.5 (1.1–2.0)	1.0 (0.7–1.4)	0.9 (0.6–1.3)	1.0 (0.6–1.7)	0.9 (0.6–1.6)
2–3 times per month	1.0 (0.7–1.6)	0.9 (0.6–1.5)	1.0 (0.6–1.7)	0.9 (0.5–1.7)	1.4 (0.7–2.6)	1.3 (0.7–2.6)
1–2 per week	1.3 (0.9–1.9)	1.3 (0.9–1.9)	0.9 (0.6–1.5)	0.8 (0.5–1.3)	1.1 (0.6–2.0)	1.0 (0.6–1.9)
3+ per week	1.3 (0.9–1.9)	1.2 (0.8–1.9)	1.1 (0.6–1.8)	1.0 (0.6–1.8)	0.8 (0.4–1.6)	0.8 (0.4–1.6)
*Muesli (moderate levels)*						
Never	1 (Ref)	1 (Ref)	1 (Ref)	1 (Ref)	1 (Ref)	1 (Ref)
Less often	0.9 (0.7–1.2)	1.0 (0.7–1.4)	0.6 (0.4–0.9)	0.7 (0.5–1.2)	0.8 (0.4–1.3)	0.8 (0.5–1.3)
2–3 times per month	1.3 (0.7–2.6)	1.3 (0.7–2.7)	0.9 (0.3–2.4)	0.9 (0.3–2.8)	1.2 (0.4–3.5)	1.2 (0.4–3.7)
1–2 per week	0.9 (0.6–1.4)	1.0 (0.7–1.6)	0.9 (0.6–1.5)	1.1 (0.6–1.8)	1.1 (0.6–1.9)	1.2 (0.6–2.2)
3+ per week	1.0 (0.7–1.4)	1.1 (0.7–1.6)	0.8 (0.5–1.3)	1.0 (0.6–1.7)	1.0 (0.6–1.7)	1.1 (0.6–2.0)
						
Various fried foods						
*Biscuits (high levels)*						
Less often/never	1 (Ref)	1 (Ref)	1 (Ref)	1 (Ref)	1 (Ref)	1 (Ref)
2–3 per month	1.2 (0.9–1.6)	1.1 (0.8–1.5)	0.9 (0.6–1.3)	1.0 (0.6–1.5)	1.0 (0.6–1.6)	1.0 (0.6–1.6)
Weekly	1.1 (0.8–1.6)	1.0 (0.7–1.4)	1.2 (0.7–1.8)	1.3 (0.8–2.1)	0.9 (0.5–1.6)	0.9 (0.5–1.7)
Daily	1.1 (0.7–1.6)	0.9 (0.6–1.4)	0.9 (0.5–1.4)	0.9 (0.5–1.7)	1.5 (0.8–2.6)	1.4 (0.7–2.6)
*Meatballs (moderate levels)*						
Never	1 (Ref)	1 (Ref)	1 (Ref)	1 (Ref)	1 (Ref)	1 (Ref)
Less often	0.6 (0.3–1.3)	0.5 (0.2–1.2)	1.7 (0.5–8.3)	0.7 (0.1–3.9)	0.5 (0.2–1.7)	0.4 (0.1–1.4)
2–3 times per month	0.7 (0.3–1.5)	0.6 (0.3–1.3)	2.3 (0.5–10.9)	0.9 (0.2–4.8)	0.6 (0.2–2.0)	0.4 (0.1–1.4)
Weekly	0.6 (0.2–1.3)	0.4 (0.2–1.1)	2.0 (0.4–10.2)	0.8 (0.1–4.8)	0.9 (0.3–3.2)	0.5 (0.2–2.0)

a Adjusted for matching factors age and gender.

b Also adjusted for smoking (current, former, never), BMI (categorical), alcohol intake (categorical-colorectal cancer only), fruit and vegetable intake (continuous), saturated fat density (quintiles, categorical), red meat density (quintiles, categorical), and total energy (log transformed).

Table 3b

**Table 3:** (continued)

	**Cancer of large bowel**		**Cancer of bladder**		**Cancer of kidney**	
	**Adjusted^a^**	**Adjusted^b^**	**Adjusted^a^**	**Adjusted^b^**	**Adjusted^a^**	**Adjusted^b^**
	**OR (95% CI)**	**OR (95% CI)**	**OR (95% CI)**	**OR (95% CI)**	**OR (95% CI)**	**OR (95% CI)**
*Breaded fish (moderate levels)*						
Never	**	**	**	**	**	**
Less often	1 (Ref)	1 (Ref)	1 (Ref)	1 (Ref)	1 (Ref)	1 (Ref)
2–3 times per month	1.2 (0.9–1.6)	1.2 (0.9–1.5)	1.4 (0.9–2.0)	1.4 (0.9–2.1)	0.9 (0.6–1.5)	0.9 (0.6–1.4)
Weekly	1.4 (1.0–2.0)	1.3 (0.9–1.9)	1.4 (0.9–2.1)	1.2 (0.7–2.0)	1.3 (0.7–2.1)	1.1 (0.6–2.0)
*Pancakes/waffles (moderate levels)*						
Never	1 (Ref)	1 (Ref)	1 (Ref)	1 (Ref)	1 (Ref)	1 (Ref)
Less often	1.6 (0.9–3.0)	1.7 (0.9–3.4)	1.0 (0.5–2.1)	1.2 (0.6–2.7)	2.0 (0.6–6.1)	2.6 (0.7–9.4)
2–3 times per month	1.4 (0.7–2.5)	1.3 (0.7–2.5)	0.8 (0.4–1.5)	0.9 (0.4–1.9)	1.5 (0.5–4.5)	1.7 (0.5–6.1)
Weekly	1.3 (0.7–2.5)	1.2 (0.6–2.3)	0.7 (0.4–1.4)	0.7 (0.3–1.6)	1.5 (0.5–4.6)	1.6 (0.4–5.9)

** No cases or controls never consumed breaded fish products.

. Controlling for potential confounders, there is little evidence of an association between any specific baked or fried potato product and cancer risk. While the data suggest a higher risk of bladder cancer for those with daily intake of any fried or baked potatoes (adjusted OR 1.6 (95% CI 0.7–3.5)), wide CIs preclude definitive assessment. There is no evidence of a positive association between crisp breads and cancer risk. Indeed, the relation between crisp bread and large bowel cancer risk appeared to be inverse (*P* for trend=0.07). No other breads or cereals were related, inversely or positively, with risk of cancer of the large bowel, bladder or kidney. With respect to other fried foods, the data suggest a small increase in risk of large bowel cancer with increasing consumption of breaded fish (*P* for trend=0.11). No other fried food items are associated with risk of the studied cancers.

The relative risk of cancer comparing quartiles of daily dietary acrylamide intake based on ranking of food items is presented in Table 4

**Table 4:** Quartiles of daily dietary acrylamide dose based on ranking of food items and risk of cancer of the large bowel, bladder and kidney

	**Cancer of large bowel**		**Cancer of bladder**		**Cancer of kidney**	
**Quartiles of ranked daily acrylamide intake**	**Adjusted^a^ OR (95% CI)**	**Adjusted^b^ OR (95% CI)**	**Adjusted^a^ OR (95% CI)**	**Adjusted^b^ OR (95% CI)**	**Adjusted^a^ OR (95% CI)**	**Adjusted^b^ OR (95% CI)**
*Overall*						
Quartile 1	1 (Ref)	1 (Ref)	1 (Ref)	1 (Ref)	1 (Ref)	1 (Ref)
Quartile 2	1.0 (0.74–1.45)	0.9 (0.6–1.3)	1.3 (0.8–2.1)	1.1 (0.7–1.8)	1.0 (0.6–1.8)	1.0 (0.6–1.9)
Quartile 3	0.9 (0.63–1.26)	0.6 (0.4–0.9)	1.0 (0.6–1.5)	0.7 (0.4–1.3)	1.2 (0.7–2.0)	1.1 (0.6–2.0)
Quartile 4	1.0 (0.71–1.42)	0.6 (0.4- 1.0)	1.2 (0.7–1.9)	0.8 (0.5–1.5)	1.0 (0.6–1.8)	0.8 (0.4–1.7)
*P*-value for trend	0.80	0.01	0.87	0.26	0.88	0.64
						
*Nonsmokers*						
Quartile 1	1 (Ref)	1 (Ref)	1 (Ref)	1 (Ref)	1 (Ref)	1 (Ref)
Quartile 2	1.0 (0.7–1.5)	0.9 (0.6–1.3)	1.4 (0.8–2.5)	1.0 (0.6–2.0)	1.0 (0.5–1.9)	1.0 (0.5–2.0)
Quartile 3	0.9 (0.6–1.3)	0.6 (0.4–0.9)	0.8 (0.5–1.6)	0.6 (0.3–1.2)	1.1 (0.6–2.1)	0.9 (0.4–1.9)
Quartile 4	1.1 (0.8–1.7)	0.6 (0.3–1.0)	1.2 (0.6–2.2)	0.7 (0.3–1.6)	1.0 (0.5–1.9)	0.7 (0.3–1.7)
*P*-value for trend	0.69	0.03	0.99	0.20	0.99	0.41
						
*Current smokers*						
Quartile 1	1 (Ref)	1 (Ref)	1 (Ref)	1 (Ref)	1 (Ref)	1 (Ref)
Quartile 2	1.1 (0.5–2.1)	1.1 (0.5–2.3)	1.2 (0.6–2.6)	1.1 (0.5–2.5)	1.3 (0.4–4.5)	1.2 (0.3–4.6)
Quartile 3	0.9 (0.4–1.9)	0.9 (0.4–2.1)	1.3 (0.6–2.8)	1.1 (0.5–2.5)	1.8 (0.5–5.9)	1.5 (0.4–6.2)
Quartile 4	0.7 (0.4–1.5)	0.7 (0.3–2.1)	1.0 (0.5–2.2)	1.0 (0.4–2.3)	1.2 (0.4–4.1)	1.3 (0.2–6.3)
*P*-value for trend	0.31	0.51	0.99	0.88	0.74	0.72

a Adjusted for matching factors age and gender.

b Also adjusted for smoking (current, former, never), BMI (categorical), alcohol intake (categorical-colorectal cancer only), fruit and vegetable intake (continuous), saturated fat density (quintiles, categorical), red meat density (quintiles, categorical), and total energy (log transformed).

. For colorectal cancer, the risk of cancer decreases with increasing quartiles of acrylamide through diet (*P* for trend=0.01). After adjusting for potential confounders, the risk of large bowel cancer for those in the highest quartile of dietary acrylamide per day was 40% lower compared to those consuming in the lowest quartile. The inverse association with large bowel cancer was evident among nonsmokers (adjusted OR=0.6 comparing highest to lowest quartile; *P* for trend=0.03) and suggestive among smokers (adjusted OR=0.7 comparing highest to lowest quartile; *P* for trend=0. 50). The relative risk between dietary acrylamide dose and cancers of the bladder and kidney was essentially null, and was similar for smokers and nonsmokers.

The findings in Table 4 were unaffected by the acrylamide concentration standard used to rank the food items, that is, using the median concentration *vs* the minimum or maximum concentration. Comparing the highest to lowest quartile, the relative risk of colorectal cancer was 0.6 using the median concentration, 0.6 using the minimum, and 0.7 using the maximum acrylamide concentration based on the NFA data. The relative risk estimates for bladder and kidney cancers were also unaffected by the acrylamide concentration used in rankings (data available on request).

## DISCUSSION

The finding in April 2002 of elevated levels of acrylamide in a variety of foodstuffs were unexpected, and led to a call by public health scientists for additional research on acrylamide (WHO, 2002). Reports from laboratory studies a few months later have provided insight into the biochemical mechanism of acrylamide formation. Acrylamide can be generated during the heating of specific foodstuffs as a result of a Maillard reaction between amino acids and sugars (Mottram *et al*, 2002; Stadler *et al*, 2002). In particular, acrylamide formation occurs when the amino acid asparagine, in the presence of sugars, is heated above 100°C. Potatoes and cereals, which had the highest measured levels of acrylamide in the Swedish NFA survey, are rich in asparagine (Belitz and Grosch, 1999).

This population-based case–control study found no association between dietary exposure to acrylamide in amounts typically ingested by Swedish adults and risk of cancers of the large bowel, bladder, or kidney. For foods with the highest acrylamide levels, namely, potato crisps, French fries, and crisp bread, there was no positive association with cancer risk. Indeed, the risk of cancer of the large bowel decreased with increasing dose of dietary acrylamide. We did observe a slightly higher risk of bladder cancer for those consuming baked or fried potatoes daily compared to never, although the CI includes the null value. Although this positive finding is in line with a previous study (Steineck *et al*, 1990), our findings overall suggest that components of potatoes other than acrylamide are responsible for this association.

The classification of acrylamide by IARC as a probable human carcinogen (IARC, 1994) was based mainly on *in vitro* and animal models. Acrylamide induces genetic mutations and chromosomal abnormalities *in vitro*, and cellular transformation *in vivo* (IARC, 1994). Long-term studies in rats and mice supported a dose–exposure relation between acrylamide and risk of cancer of the lung, mammary gland, thyroid, oral cavity, and intestinal and reproductive tract (Bull *et al*, 1984; Johnson *et al*, 1986). Moreover, animals administered acrylamide orally (Paulsson *et al*, 2002) or fed a diet high in fried foods (Tareke *et al*, 2000) had higher levels of haemoglobin DNA adducts compared to unexposed animals.

The human data were less clear and limited to occupational settings. In a small cohort of 371 workers exposed to acrylamide through organic dyes, cancer mortality was higher than expected, mainly because of deaths from cancer of the digestive tract and respiratory system (Sobel *et al*, 1986). More recently, Marsh *et al*, (1999) in a cohort of 8500 workers with potential occupational exposure found little evidence for an excess risk of cancer mortality overall. While an excess of thyroid cancer and a dose–exposure relation with pancreatic cancer were suggested, the wide CIs precluded definitive assessments. The study had greater power to detect an effect of lung cancer, which showed minimal excess risk between both workers exposed and unexposed to acrylamide compared to the general population. Among 200 construction workers exposed to high levels of acrylamide for 20 months, 80% had haemoglobin adduct levels above the normal background range (Hagmar *et al*, 2001).

One interpretation of our null finding is that no association between dietary acrylamide and cancer risk exists, implying that species differences negate extrapolating from experimental animals to humans, as shown for other carcinogens (IARC, 1987). In addition, human intake of dietary acrylamide is several folds lower than doses tested in animal experiments. Acrylamide intake within the range of human exposure may thus be effectively detoxified. Although a comprehensive coverage of dietary acrylamide was not possible, certain aspects of our study would support the validity of its negative findings. It was large and population-based with reasonably high response rates, thus reducing the possibility of selection bias while the data on demographic, lifestyle, and dietary, covariates would have reduced the opportunity for confounding in the analysis.

Could certain limitations and possible biases have attenuated a true positive association? First, the acrylamide content of a number of food items has not yet been characterised, so our values of daily intake of dietary acrylamide may be underestimated. Measurement errors of acrylamide intake in cases and controls would entail such attenuation (Rothman and Greenland, 1998). There was also variability in acrylamide dose across brands of a given food. It is relevant that it is ranking of individuals with respect to exposure, rather than absolute intake, that determines the calculated relative risk in case–control studies. In fact, when comparing an abbreviated *vs* extensive food frequency questionnaire, increasing the number of food items improves the ranking, and thereby the relative risk, only to a small degree (Voskuil *et al*, 1999). In our own data, we found the relative risk estimates comparing quartiles of total acrylamide dose to be insensitive to the concentration of acrylamide used to rank individual food items. Although we possibly capture only partial intake of acrylamide, it is likely that we have a ranking that is valid for drawing conclusions.

Second, a true association may be concealed if the level of exposure in the studied population is low and/or if the range of variation is limited. Of the food items found to contain the highest levels of acrylamide, only biscuits and pan-fried potatoes were commonly consumed by this population. Risk assessment models for humans suggest that lifetime risk for cancer is 0.7–4.5 per 1000 based on consumption of 1 *μ*g acrylamide per kg body weight per day (US EPA, 1985; WHO, 1985). In our study, less than 2% of the population was estimated to intake acrylamide through diet at these levels. Acrylamide intake through dietary sources may thus be effectively detoxified within the range of human exposure. This hypothesis is suggested for heterocyclic amines, which cause cancer when given to rodents, but does not appear to in humans, where doses are typically millionth of those given in studies of animal carcinogenicity (Augustsson *et al*, 1999).

Third, residual confounding is a possible explanation for some of our findings, such as the inverse association between total acrylamide dose and large bowel cancer. The food items under study contain a multitude of nutrients. Although we have controlled analytically for total energy, saturated fat, meat, and fruits and vegetables, it may be difficult to disentangle the protective effect of specific nutrients from that of acrylamide. Lastly, although no excess risk was observed for the three major cancers studied, we cannot rule out a possible excess risk of other cancer sites. However, large bowel, bladder, and kidney would be likely target sites, because acrylamide and its metabolite glycidamide are detoxified by glutathione conjugation, are water soluble, and are absorbed quickly in the digestive tract and excreted via the urine (IARC, 1994).

This first study of dietary acrylamide in relation to three major human cancers is reassuring. Needless to say, additional epidemiological evidence is required, notably for other cancer sites as well as for neurological and other disorders. While the null hypothesis of no effect can never be scientifically proven, it would be useful to determine cooking methods that avoid acrylamide formation during food preparation. Since measuring error in acrylamide intake would entail underestimation of any true association with cancer risk, validation studies of acrylamide dose using existing food questionnaires should be a high priority.
